# Traumatic Brain Injury and Dementia in Medicare Population: Differences in Risk Between Veterans and Non-Veterans

**DOI:** 10.1093/geroni/igaa057.3123

**Published:** 2020-12-16

**Authors:** Arseniy Yashkin

**Affiliations:** Duke University, Durham, North Carolina, United States

## Abstract

The aim of this study was to assess differences in the effect of traumatic brain injury (TBI) on the onset of Alzheimer’s disease (AD) and other dementias between veteran and non-veteran respondents of the Health and Retirement Study as well as to measure the sensitivity of these differences to the introduction of controls for groups of demographic, medical co-morbidity and polygenic risk scores reflecting AD hallmarks. Using the Fine-Gray proportional hazards model we found that TBI was a strong predictor of dementia in community dwelling residents age 65+: for AD associated risk was 181% [Hazard Ratio (HR): 2.81; CI:2.05-3.86] sample-wide and 142% [HR: 2.42; CI:1.31-2.46] in veteran males. Effect magnitude decreased with the addition of risk-related control variables but remained associated with significantly increased risk. Large differences in risk were observed between veteran and non-veteran males for AD, vascular dementia, senile dementia, and dementia with Lewy Bodies

